# Bounds and Inequalities Relating *h*-Index, *g*-Index, *e*-Index and Generalized Impact Factor: An Improvement over Existing Models

**DOI:** 10.1371/journal.pone.0033699

**Published:** 2012-04-04

**Authors:** Ash Mohammad Abbas

**Affiliations:** Department of Computer Engineering, Aligarh Muslim University, Aligarh, India; University of Illinois-Chicago, United States of America

## Abstract

In this paper, we describe some bounds and inequalities relating 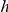-index, 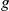-index, 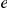-index, and generalized impact factor. We derive the bounds and inequalities relating these indexing parameters from their basic definitions and without assuming any continuous model to be followed by any of them. We verify the theorems using citation data for five Price Medalists. We observe that the lower bound for 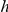-index given by Theorem 2, 
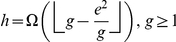
, comes out to be more accurate as compared to Schubert-Glanzel relation 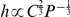 for a proportionality constant of 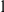, where 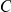 is the number of citations and 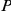 is the number of papers referenced. Also, the values of 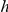-index obtained using Theorem 2 outperform those obtained using Egghe-Liang-Rousseau power law model for the given citation data of Price Medalists. Further, we computed the values of upper bound on 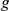-index given by Theorem 3, 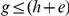, where 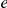 denotes the value of 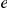-index. We observe that the upper bound on 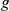-index given by Theorem 3 is reasonably tight for the given citation record of Price Medalists.

## Introduction

A lot of research is carried out by people working in different areas. Sometimes, one needs to evaluate the quality of the research produced by individual authors or groups of authors. The quality of research produced by authors is, generally, evaluated in terms a ranking parameter which is, generally, based on the number of citations received by the papers produced by the authors. There are many types of ranking parameters presented in the literature for evaluating the quality of research such as 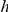-index [1], 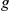-index [2], 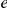-index [3], and impact factor [4]. The impact factor in the long term becomes the average number of citations per paper. This long term impact factor is termed as the *generalized impact factor*.

While one has computed an index for evaluating the quality of research, one would like to get an indication about the other types of indices. To have such an indication, one needs to know how an index is related to other indices. The relationships among 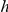-index, 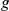-index, and 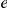-index are described in [5]. However, in [5], the indices are assumed to follow a continuous distribution. A relation between 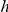-index and impact factor is described in [6] using a power law model called the Lotka's model.

In this paper, we describe the bounds for the 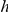-index and 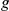-index in terms of the indices and the generalized impact factor. We derive these bounds from the very basic definitions of the indices and the generalized impact factor without assuming any model or any continuous distribution to be followed by any of these indices. We verify the theorems for citation records of five Price Medalists. Also, we compare the values of 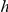-index with those obtained using Schubert-Glanzel formula and Egghe-Liang-Rousseau's power law model. Further, we discuss the tightness of the upper bound on 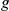-index for Price-Medalists.

In what follows, we present an analysis of the indices and the generalized impact factor.

## Analysis

In this section, we wish to analyze the relationships among the indices and the generalized impact factor. To do so, we first present an overview of the indices and the generalized impact factor, and then we shall analyze the relationships among them.

### Overview of Indices and Impact Factor

In this subsection, we briefly define the generalized impact factor and different types of indices.

#### The 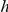-Index

Suppose the papers are arranged in descending order of the number of citations. Let 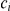 be the number of citations of a paper numbered 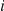. The 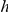-index [1], when papers are arranged in descending number of their citations, can be defined as follows.
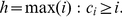
(1)By definition, 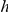-index is the largest number, 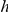, such that the papers arranged in their decreasing order of citations have at least 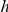 number of citations.

#### The 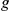-Index

According to the definition of 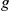-index, if the papers are arranged in the descending order of their number of citations, 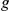 is the largest number such that the summation of the number of citations is at least 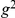. In other words, when papers are arranged in descending order of their citations, 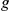-index can be defined as follows.
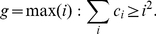
(2)Note that 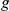-index is the largest number 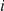 such that 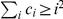.

#### The 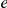-Index

The 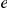-index is defined in [3] to serve as a complement for the 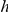-index. The definition of 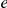-index is as follows.
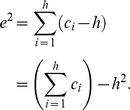
(3)Alternatively, (3) can be written as follows.
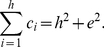
(4)


*Remark:* In the definitions of 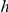-index (as given by (1)) and that of 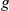-index (as given by (2)), we have intentionally ignored the time 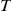 at which we are considering their values. This is done to keep their definitions simple, and defining so there is no loss of generality as far as the discussion in this work is concerned. For precise definitions of the indices incorporating the time, one is referred to [7]. The same is true for the 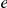-index. Secondly, while defining the indices and the impact factor, we assume that the number of papers, 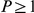, and the numbers of citations received by *i*th paper, 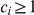. This is also true for the theorems proved in this paper.

#### Generalized Impact Factor

Let 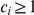 be the number of citations of the paper numbered 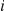, and let 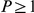 be the number of papers. The *generalized impact factor* is defined as follows.
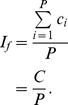
(5)Note that the *generalized impact factor* is simply called *impact factor* in [6]. We have added the prefix “generalized” to differentiate it from the impact factor that uses a time window constraint. Actually, the impact factor given by (5) (and also that given in [6]) denotes the average number of citations received per paper.

### Analysis of Relationships

In this subsection, we describe how indices and generalized impact factor are related to one another.

#### Impact Factor, 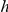-Index and 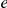-Index

We state the following theorem that relates these parameters.

**Theorem 1** *Let* 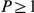 *be the number of papers and let* 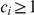 *be the numbers of citations received by ith paper. The* 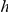 *-index,* 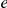 *-index and impact factor are related by the following inequality*.
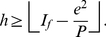
(6)


*Proof*. Using (5), the total number of citations can be written as follows.
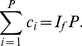
(7)The citations appearing in the L.H.S. of (7) can be broken into two parts, one from 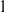 to 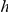 and the other from 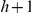 to 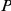, as given below.
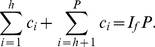
(8)Using (4) and (8), we have,
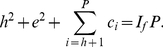
(9)Now, we have,
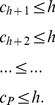
(10)Therefore, we have,
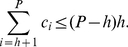
(11)Using (9) and (11), we have,
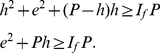
(12)In other words, we have,
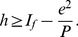
(13)Since 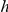 is a whole number, therefore, we can write,
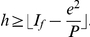



In other words, we can say that
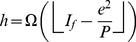
(14)where, 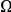 denotes the lower bound. For definitions of different types of bounds, we refer the readers to [8].

#### The 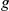-Index, 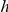-Index, and 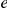-Index

We state the following theorem that provides an inequality relating these indices.

**Theorem 2** *The* 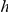 *-index,* 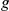 *-index, and* 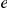 *-index are related by the following inequality*.
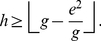
(15)


*Proof*. Let the the papers are arranged in the descending order of their citations. From the definition of 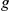-index, as given in (2), we have,
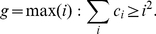
(16)At 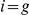, we have,
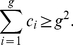
(17)Breaking the number of citations in the L.H.S. of (17) into parts, we have,
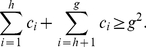
(18)Using (4) and (18), we have,
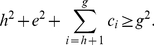
(19)In other words,
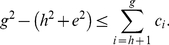
(20)Now, we have,
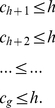
(21)Therefore, we have,
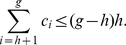
(22)Using (20) and (22), we have,
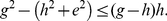
(23)Or,
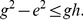
(24)Rearranging (24), we have,
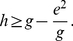
(25)Since all these indices, 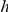, 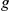, and 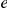 are integers, therefore, (25) can be written as follows.
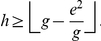



In other words, Theorem 2 provides a lower bound for 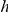-index in terms of the 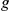-index and the 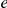-index.
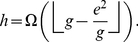
(26)We have the following lemma that provides a bound for the 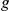-index.

**Lemma 1**. *An upper bound for* 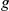 *-index is as follows*.
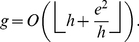
(27)


*Proof*. From (20), we have,
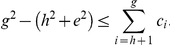
In (21), if we put 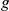 at the R.H.S. for 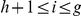, 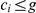, we get,
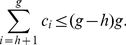
(28)Therefore, from (20), we have,
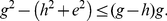
(29)Or,
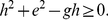
(30)Or,
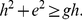
(31)This gives us,
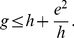
(32)Again, all these indices are whole numbers, therefore, we can write,
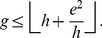
(33)Alternatively,
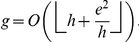



We now prove another theorem that provides an upper bound for the 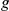-index in terms of 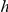-index and 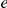-index.

**Theorem 3**. *An upper bound for* 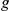 *-index in terms of* 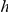 *-index and* 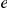 *-index is as follows*.
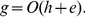
(34)


*Proof*. Using (24), we have,
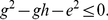
(35)This resembles to the quadratic equation 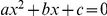, whose roots are as follows.
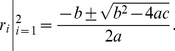
(36)Here, we have, 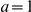, 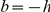, 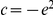, therefore, the only root for 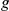-index is,
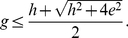
(37)Now, we know that 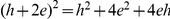. In other words, we have,
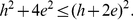
(38)This implies that
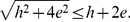
(39)Using (37) and (39), we have,
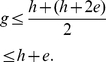
(40)In other words, 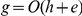.

#### The 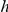-Index, 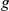-Index, and Impact Factor

We state the following theorem that relates these parameters.

**Theorem 4**. *The generalized impact factor,* 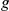 *-index, and* 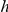 *-index are related as per the following inequality*.
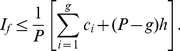
(41)


*Proof*. From (5), we have,
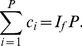
(42)Breaking the number of citations in the L.H.S. of (42), we have,
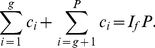
(43)Now, we have,
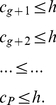
(44)Therefore, we have,
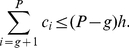
(45)Using (43) and (44), we have,
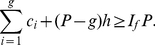
(46)Or,
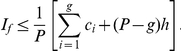



In other words, Theorem 4 states an upper bound for the generalized impact factor which is as follows.
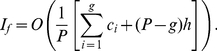
(47)


#### Utility of Bounds

We wish to point out that lower and upper bounds are very common in the area of Computer Science and Engineering. They are useful when either one cannot find exact expressions or it is difficult to derive the exact expressions. Using the bounds, one can say that the parameter lies above it (for a lower bound) or below it (for an upper bound). To the best of our knowledge, the exact relationships among the 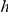-index, 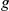-index, 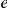-index, and impact factor have not been described by any researcher till date. In the absence of such exact expressions, we suggest to use the lower and upper bounds, and it forms the motivation behind the derivation of bounds and inequalities presented in this paper. In our view, one can realize where the value of an indexing parameter lies given another set of parameter(s) without going through the whole citation database (of an author, a journal, an institution, a country or a region).

### Existing Relationship Models

In this subsection, we briefly describe the existing models that relate some of the indices.

#### Schubert-Glanzel Formula

Let 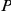 be the number of papers referenced and 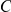 be the number of citations. According to Schubert-Glanzel model [9], the 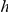 index is given by the following expression.
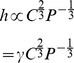
(48)where, 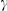 is a proportionality constant. Another form of Schubert-Glanzel formula is,
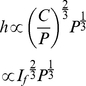
(49)which is equivalent to that given by (48), however, (49) is in terms of the generalized impact factor.

The major drawback of Schubert-Glanzel formula is that it does not say anything about the value of the proportionality constant. In [10], the proportionality constant 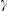 is assumed to be 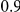 for journals and 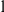 for other sources. In the absence of a specific value of the proportionality constant, we assume it to be equal to 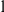.

#### Egghe-Liang-Rousseau Model

A relationship between 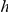-index and generalized impact factor, 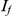, is presented by Egghe, Liang and Rousseau in [6], which is based on power law model and is as follows.
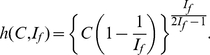
(50)Since 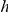-index is an integer, therefore, it is better to consider the ceiling of the R.H.S. of (50). In [6], it has been argued that when 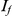 tends to 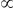, 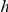 tends to 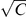.

In what follows, we verify the theorems and lemma proved in the previous section and compare them with the existing models.

## Results and Discussion

In this section, we first verify our theorems using citation data for a set of scientists, for example, a set of five Price Medalists, and then compare them with the existing models. We collected the citation data for the given set of Price Medalists using *scHolar index* [11], which is based on Google Scholar. The numbers of citations of each referenced paper of Price Medalists are given in Medalist S1, S2, S3, S4, and S5.

Table 1 shows the number of citations (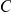), the number of papers referenced (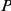), 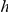-index, 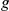-index, and generalized impact factor (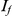) for Price Medalists as per the citation data given in Medalist S1, S2, S3, S4, and S5. The values of 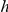-index, 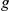-index, and generalized impact factor shown in Table 1 are the actual values. In what follows, we verify the theorems for Price Medalists.

**Table 1:** The number of citations (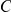), number of papers referenced (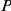), 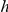-index, 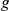-index, and generalized impact factor (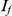) for a set of five Price Medalists.

*Price Medalists*	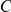	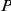	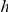 *-Index*	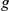 *-Index*	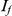
Medalist S1	12674	520	45	101	24.37
Medalist S2	4861	180	38	62	27.01
Medalist S3	2701	110	30	48	24.55
Medalist S4	3556	176	27	54	20.20
Medalist S5	2785	130	26	48	21.42

### Verification of Theorems

Table 2 shows a verification of Theorems for Price Medalists. The first row of the table shows the statements of each theorem and lemma. The symbol 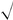 under the bound shows that the given theorem is verified. For example, consider Medalist S1 for whom 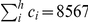, and therefore, 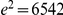. Theorem 1 gives 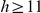, and the value of 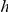-index for Medalist S1 is 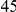. Since 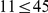, therefore, Theorem 1 is verified. Theorem 2 gives 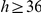, which is less than 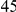, therefore, Theorem 2 also is verified. Lemma 1 gives 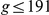, and the value of 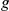-index for Medalist S1 is 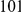. Since 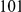 is less than 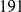, therefore, Lemma 1 is verified. Theorem 3 gives 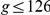, and since 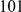 is less than 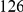 therefore, Theorem 3 is verified. For verification of Theorem 4, we have, 
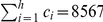
, and 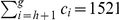. Therefore, 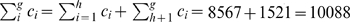. Theorem 4 gives 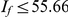, and since 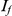 for Medalist S1 is 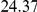, which is smaller than 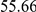, therefore, Theorem 4 is verified. Similarly, we can verify the theorems and lemma proved in this paper for other Price Medalists. The supplement data in terms of the values of intermediate parameters needed to verify the theorems and lemma is shown in Table 3.

**Table 2:** Verification of theorems for the given set of Price Medalists.

*Price*	*Theorem 1*	*Theorem 2*	*Lemma 1*	*Theorem 3*	*Theorem 4*
*Medalists*	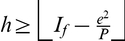	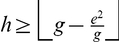	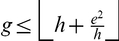	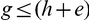	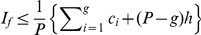
Medalist S1	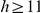	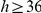	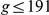	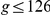	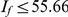
	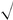	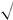	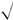	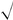	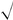
Medalist S2	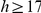	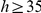	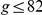	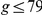	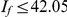
	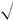	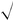	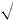	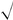	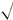
Medalist S3	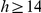	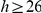	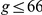	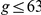	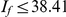
	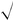	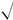	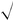	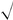	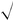
Medalist S4	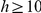	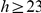	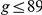	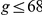	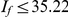
	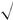	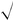	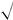	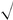	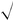
Medalist S5	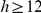	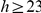	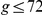	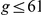	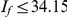
	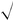	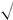	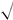	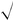	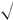

**Table 3:** The supplemental data in terms of intermediate parameters for the given set of Price Medalists.

*Price Medalists*	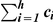	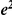	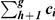	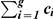
Medalist S1	8567	6542	1521	10088
Medalist S2	3085	1641	740	3825
Medalist S3	1956	1056	409	2365
Medalist S4	2381	1652	524	2905
Medalist S5	1855	1179	453	2308

### Tightness of Bounds

Note that there are two lower bounds for 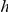-index, the one given by Theorem 1 and the other given by Theorem 2. Using Table 2, we see that the lower bound on 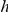-index given by Theorem 2 is closer to the actual values as compared to that given by Theorem 1. Similarly, there are two upper bounds for 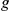-index, the one given by Lemma 1 and the other given by Theorem 3. We observe from Table 2 that the upper bound on 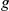-index given by Theorem 3 is closer to the actual values of 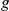-index as compared to those given by Lemma 1. In other words, the bounds given by Theorem 2 and Theorem 3 are more tight as compared to those given by Theorem 1 and Lemma 1, respectively.

Table 4 shows the actual values of 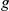-index and the values of 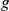-index obtained using Theorem 3. Also, we computed the errors in the values given by Theorem 3 as compared to the actual values of 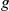-index for Price Medalists. We observe that the upper bound on the 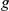-index given by Theorem 3 is reasonably tight.

**Table 4:** Errors in the 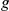-index using Theorem 3 for the given set of Price Medalists.

*Price Medalists*	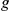 *-index*	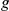 *-index (Theorem 3)*	
		Value	Error(%)
Medalist S1	101	126	24.75
Medalist S2	62	79	27.41
Medalist S3	48	63	31.25
Medalist S4	54	68	25.92
Medalist S5	48	61	27.08

### Improvements over Schubert-Glanzel and Egghe-Liang-Rousseau Models

We computed the 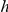-index using Theorem 2. Also, we computed the values of 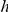-index for Price Medalists using Schubert-Glanzel formula given by (48) and using Egghe-Liang-Roussea's power law model given by (50). Note that the values of 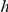-index using any of these three models are approximate values. To study closeness of these approximate values to the exact values, we computed the percentage errors in the approximate values of 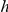-index with respect to the exact values, which are shown in Table 5. We observe that the percentage error in case of the values obtained using Theorem 2 is significantly less as compared to those obtained using either Schubert-Glanzel formula or Egghe-Liang-Rousseau power law model. For example, for Medalist S1, the exact value of 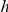-index is 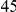, the lower bound given by Theorem 2 is 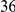. The values of 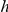-index obtained using Schubert-Glanzel formula is 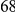 and that obtained using Egghe-Liang-Rousseau's power law model is 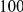. The error using Theorem 2 is 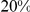 and the error in the value obtained using Schubert-Glanzel formula is 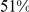. The error in the value of 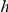-index using Egghe-Liang-Rousseau's model is 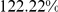. Similarly, one can see from Table 5 that Theorem 2 provides a significant improvement over both Schubert-Glanzel formula and Egghe-Liang-Roussea's power law model.

**Table 5:** Errors in the 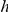-index using Theorem 2, Schubert-Glanzel model, and Egghe-Liang-Rousseau's power law model [6] for the given set of Price Medalists.

*Price Medalists*	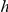 *-Index*	*Lower Bound*		*Schubert-Glanzel*		*Egghe et al*	
		Value	Error(%)	Value	Error(%)	Value	Error(%)
Medalist S1	45	36	20.00	68	51.11	100	122.22
Medalist S2	38	35	7.89	51	34.21	64	68.42
Medalist S3	30	26	13.33	41	36.66	47	56.66
Medalist S4	27	23	14.81	42	51.85	53	96.30
Medalist S5	26	23	11.54	40	53.85	47	80.77

### Conclusion

Finding the relationships among indexing parameters for determining the quality of research is a challenging task. In this paper, we described some inequalities relating 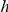-index, 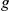-index, 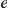-index, and generalized impact factor. We derived the inequalities from the very basic definitions of these indexing parameters and without assuming any continuous model to be followed by any of them. However, the relationships in the form of bounds and inequalities among the indices are not trivial, and to the best of our knowledge, we are the first ones to present such kinds of relationships.

We verified the theorems and lemma presented in this paper for citation records of Price Medalists. We observed that the lower bound on 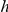-index given by Theorem 2 is more tight as compared to that given by Theorem 1. The upper bound on 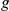-index given by Theorem 3 is more tight as compared to that given by Lemma 1.

We compared the values of 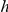-index obtained using Theorem 2 with the values of 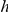-index obtained using either Schubert-Glanzel formula or Egghe-Liang-Rousseau model. We observed that the values of 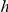-index obtained using Theorem 2 are significantly closer to the exact values as compared to those obtained using either Schubert-Glanzel formula or Egghe-Liang-Rousseau's power law model. This enables us to conclude that Theorem 2 provides significant improvements over both Schubert-Glanzel formula as well as Egghe-Liang-Rousseau's model.

Further, we computed the upper bound given by Theorem 3 which states that 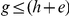, where 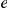 denotes the 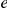-index. We observed that the upper bound on 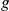-index given by Theorem 3 is reasonably tight for the given citation record of Price Medalists. In future, one may propose more tight bounds for either 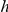-index or 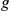-index.

## Supporting Information

Medalist S1 **Citation data for Price Medalist 1 using scHolar index** [**11**] **, which is based on Google Scholar. Includes the numbers of citations of each referenced paper of Price Medalist 1.**
(DOC)

Medalist S2 **Citation data for Price Medalist 2 using scHolar index** [**11**] **, which is based on Google Scholar. Includes the numbers of citations of each referenced paper of Price Medalist 2.**
(DOC)

Medalist S3 **Citation data for Price Medalist 3 using scHolar index** [**11**] **, which is based on Google Scholar. Includes the numbers of citations of each referenced paper of Price Medalist 3.**
(DOC)

Medalist S4 **Citation data for Price Medalist 4 using scHolar index** [**11**] **, which is based on Google Scholar. Includes the numbers of citations of each referenced paper of Price Medalist 4.**
(DOC)

Medalist S5 **Citation data for Price Medalist 5 using scHolar index** [**11**] **, which is based on Google Scholar. Includes the numbers of citations of each referenced paper of Price Medalist 5.**
(DOC)
