# Supplementary material for: Bounds and Inequalities Relating h-Index, g-Index, e-Index and Generalized Impact Factor: An Improvement over Existing Models
Source: PLoS One. 2012 Apr 4;7(4):e33699. doi: 10.1371/journal.pone.0033699 (PMC3319552; doi:10.1371/journal.pone.0033699)
Supplement: Medalist S1 — Citation data for Price Medalist 1 using scHolar index [11] , which is based on Google Scholar. Includes the numbers of citations of each referenced paper of Price Medalist 1. (DOC) [file pone.0033699.s001.doc]

We collected the citation data for five Price Medalists using *scHolar index* [11], which is based on Google Scholar. In what follows, we provide the numbers of citations of each referenced paper of Price Medalists.

**Medalist S1.**

Referenced Papers: 520, Citations: 12674, Citations/Reference: 24.37, h-index: 45, g-index: 101. The numbers of citations of each paper in descending order are as follows.

1249, 1127, 904, 621, 357, 328, 242, 232, 180, 177, 171, 167, 153, 151, 135, 131, 128, 127, 123, 115, 108, 102, 100, 95, 94, 93, 88, 88, 75, 74, 72, 67, 65, 65, 60, 59, 57, 55, 52, 50, 48, 46, 46, 45, **45***^h^*, 45, 44, 44, 44, 43, 43, 41, 40, 40, 39, 38, 37, 36, 33, 33, 30, 30, 30, 28, 27, 26, 26, 26, 26, 26, 26, 26, 24, 24, 24, 23, 23, 22, 22, 22, 22, 22, 21, 21, 21, 21, 21, 21, 20, 20, 20, 19, 19, 18, 18, 18, 18, 18, 18, 17, **17***^g^*, 17, 17, 16, 16, 16, 16, 16, 16, 16, 16, 16, 15, 15, 15, 15, 15, 15, 15, 15, 15, 14, 14, 14, 14, 14, 14, 14, 14, 14, 14, 13, 13, 13, 13, 13, 13, 13, 13, 13, 12, 12, 12, 12, 12, 12, 12, 12, 12, 12, 12, 12, 12, 12, 12, 12, 12, 11, 11, 13 11, 11, 11, 11, 11, 11, 11, 11, 11, 11, 11, 11, 11, 11, 10, 10, 10, 10, 10, 10, 10, 10, 10, 10, 10, 10, 10, 9, 9, 9, 9, 9, 9, 9, 9, 9, 9, 9, 9, 9, 9, 9, 9, 9, 9, 9, 9, 9, 9, 8, 8, 8, 8, 8, 8, 8, 8, 8, 8, 8, 8, 8, 8, 8, 8, 8, 7, 7, 7, 7, 7, 7, 7, 7, 7, 7, 7, 7, 7, 7, 7, 7, 7, 7, 7, 7, 7, 7, 7, 7, 7, 7, 7, 6, 6, 6, 6, 6, 6, 6, 6, 6, 6, 6, 6, 6, 6, 6, 6, 6, 6, 6, 6, 5, 5, 5, 5, 5, 5, 5, 5, 5, 5, 5, 5, 5, 5, 5, 5, 5, 5, 5, 5, 5, 5, 5, 5, 5, 5, 5, 5, 5, 5, 5, 5, 5, 4, 4, 4, 4, 4, 4, 4, 4, 4, 4, 4, 4, 4, 4, 4, 4, 4, 4, 4, 4, 4, 4, 4, 4, 4, 4, 4, 4, 4, 4, 4, 4, 4, 4, 4, 4, 4, 4, 4, 4, 4, 4, 4, 4, 4, 4, 4, 4, 3, 3, 3, 3, 3, 3, 3, 3, 3, 3, 3, 3, 3, 3, 3, 3, 3, 3, 3, 3, 3, 3, 3, 3, 3, 3, 3, 3, 3, 3, 3, 3, 3, 3, 3, 3, 3, 3, 3, 3, 3, 3, 3, 3, 3, 3, 3, 3, 3, 2, 2, 2, 2, 2, 2, 2, 2, 2, 2, 2, 2, 2, 2, 2, 2, 2, 2, 2, 2, 2, 2, 2, 2, 2, 2, 2, 2, 2, 2, 2, 2, 2, 2, 2, 2, 2, 2, 2, 2, 2, 2, 2, 2, 2, 2, 2, 2, 2, 2, 2, 2, 2, 2, 2, 2, 2, 2, 2, 2, 2, 2, 2, 2, 2, 2, 2, 2, 2, 2, 2, 2, 2, 2, 2, 2, 2, 2, 2, 2, 2, 2, 2, 2, 2, 2, 2, 1, 1, 1, 1, 1, 1, 1, 1, 1, 1, 1, 1, 1, 1, 1, 1, 1, 1, 1, 1, 1, 1, 1, 1, 1, 1, 1, 1, 1, 1, 1.
