# Supplementary material for: Bounds and Inequalities Relating h-Index, g-Index, e-Index and Generalized Impact Factor: An Improvement over Existing Models
Source: PLoS One. 2012 Apr 4;7(4):e33699. doi: 10.1371/journal.pone.0033699 (PMC3319552; doi:10.1371/journal.pone.0033699)
Supplement: Medalist S5 — Citation data for Price Medalist 5 using scHolar index [11] , which is based on Google Scholar. Includes the numbers of citations of each referenced paper of Price Medalist 5. (DOC) [file pone.0033699.s005.doc]

**Medalist S5.**

Referenced Papers: 130, Citations: 2785, Citations/Reference: 21.42, h-index: 26, g-index: 48. The numbers of citations of each paper in descending order are as follows.

166, 153, 149, 142, 141, 112, 103, 84, 77, 69, 62, 57, 51, 49, 43, 42, 42, 40, 38, 37, 36, 36, 35, 33, 31, **27***^h^*, 26, 26, 25, 25, 25, 24, 24, 23, 22, 22, 21, 20, 20, 19, 18, 18, 17, 17, 16, 15, 15, **15***^g^*, 14, 14, 13, 12, 12, 12, 12, 12, 12, 11, 11, 11, 11, 10, 10, 10, 10, 9, 9, 8, 8, 8, 8, 8, 8, 7, 7, 7, 7, 7, 7, 7, 6, 6, 6, 6, 6, 6, 6, 6, 5, 5, 5, 5, 5, 5, 4, 4, 4, 4, 4, 4, 4, 4, 3, 3, 3, 3, 3, 3, 3, 3, 3, 2, 2, 2, 2, 2, 2, 2, 2, 2, 1, 1, 1, 1, 1, 1, 1, 1, 1, 1.
